# COVID-19-Associated Disseminated Intravascular Coagulopathy Presenting As Inferior ST-Segment Elevation Myocardial Infarction

**DOI:** 10.7759/cureus.39308

**Published:** 2023-05-21

**Authors:** Narek Hakobyan, Nosakhare Ilerhunmwuwa, Mustafa Wasifuddin, Anika Tasnim, Avezbakiyev Boris

**Affiliations:** 1 Internal Medicine, Brookdale University Hospital Medical Center, Brooklyn, USA; 2 Hematology/Oncology, Brookdale University Hospital Medical Center, Brooklyn, USA

**Keywords:** inferior wall myocardial infarction, coagulapathy, covid-19, st-elevation myocardial infarction (stemi), disseminated intravascular coagulopathy

## Abstract

Disseminated intravascular coagulopathy (DIC) is infrequently associated with COVID-19 infection. COVID-19 infection can predispose to thrombotic events through inflammation and microvascular injury. DIC is rarely associated with coronary artery disease, especially myocardial infarction (MI). In this case report, we present an uncommon case of a patient with concurrent DIC and MI in the setting of COVID-19 infection. A 73-year-old male patient with no known cardiovascular risk factor presented with syncope. Assessment in the field by emergency medical service (EMS) showed the patient had a third-degree atrioventricular block and a heart rate of 40 beats per minute. He was given atropine and transcutaneously paced. Upon admission, he was found to have an inferior wall ST-elevation myocardial infarction (STEMI) and tested positive for COVID-19. Cardiac catheterization was performed urgently and revealed triple vessel disease. Attempts to revascularize the vessels were unsuccessful. He subsequently developed cardiogenic shock. He was started on multiple pressor support. Laboratory workup was suggestive of DIC, and he later developed multiorgan failure. Continuous renal replacement therapy was initiated but failed due to persistent thrombosis of the dialysis access. Despite all measures, the patient developed cardiac arrest and passed away on the third day of hospitalization. Our understanding of COVID-19 and its complications has grown exponentially since the beginning of the pandemic. The pro-inflammatory state induced by the disease creates a hypercoagulable state that may result in thrombotic complications, including MI. In severe cases, a consumptive coagulopathy may develop, leading to DIC. This unique case report seeks to highlight the importance of staying vigilant about the potential complications of MI and DIC induced by COVID-19 so that prompt diagnosis can be made to reduce morbidity and mortality in these patients.

## Introduction

The novel COVID-19 was declared a pandemic by the WHO in March 2020. Globally, more than six million people have died from the disease. In addition to conventional respiratory and constitutional symptoms, cardiovascular manifestations, such as thrombotic events, are common. These events include venous thromboembolism, cerebrovascular accidents, and acute myocardial infarctions (MIs) [1-4]. Thrombosis occurs due to complement-mediated microvascular injury, a systemic response that generates procoagulants, and the release of cytokines that set off the coagulation cascade [5-7]. Furthermore, the systemic inflammatory response contributes to developing thrombosis by generating procoagulants [7]. It is also possible for cytokines to be released secondary to other means exposing tissue factors and causing coagulation. These thrombotic events, when severe, can eventually lead to disseminated intravascular coagulation (DIC) [8]. COVID-19 can be associated with DIC, although this is relatively uncommon [9-13]. DIC results in systemic thrombosis but rarely involves the coronary vessels [11,14-17]. A limited number of reports in the literature describe COVID-19-associated DIC presenting as ST-elevation myocardial infarction (STEMI) [18, 19]. Herein, we present another instance of this association to emphasize the importance of maintaining a high index of suspicion and the challenges associated with managing patients in this situation.

## Case presentation

A 73-year-old male with a medical history significant for osteoarthritis and kidney stones was transported by ambulance to the ED following a sudden loss of consciousness. A preliminary assessment by the emergency medical services (EMS) revealed that the patient was unconscious and had bradycardia with a heart rate of 42 bpm, a third-degree heart block, and ST-segment elevation in leads II, III, and arteriovenous fistulas (AVFs) on an EKG. Atropine was administered immediately, followed by transcutaneous pacing at a heart rate of 80 bpm with successful capture, and subsequently, the patient was intubated. Intracranial pathology was ruled out with a CT scan of the head in the ED. The laboratory test results are shown in Table 1.

**Table 1:** Laboratory results. pBNP: Pro B-type natriuretic peptide; PT: Platelets; INR: International normalised ratio; PTT: Partial thromboplastin time; HDL: High-density lipoprotein; LDL: Low-density lipoprotein; HBG: Hemoglobin; HCT: Hematocrit; MCV: Mean corpuscular volume.

Laboratory markers	Normal values	Day 1	Day 2
Troponin I	< = 0.034 ng/mL	15.400	28.700
N-Terminal pBNP	11.1-125.0 pg/mL	13,800	-
Creatine Kinase	55.0-170.0 U/L	490.0	734.0
PT	9.2-12.8 sec	37.1	43.2
INR	0.70-1.20	3.27	3.81
PTT	23.5-35.5 sec	41.1	43.4
Fibrinogen	250.0-520.0 mg/dL	95.0	81.0
D-Dimer	< = 230 ng/mL DDU	13,116	-
Cholesterol	120-200 mg/dL	88	-
Triglycerides	<150 mg/dL	135	-
HDL	60-80 mg/dL	21.0	-
LDL	50-130 mg/dL	40	-
Chol/HDL Ratio	2.80-4.80	4.19	-
WBC	4.1-10.1 10x3/uL	16.1	15.7
RBC	4.33-5.43 10x6/uL	3.48	3.19
HBG	12.9-16.7 g/dL	11.5	9.0
HCT	40-47 %	29.6	27.4
MCV	80.8-94.1 fL	84.8	85.8
Platelets	153-328 10x3/uL	64	69
Hemoglobin A1C	4.8-5.6 %	7.3	-

**Figure 1:**
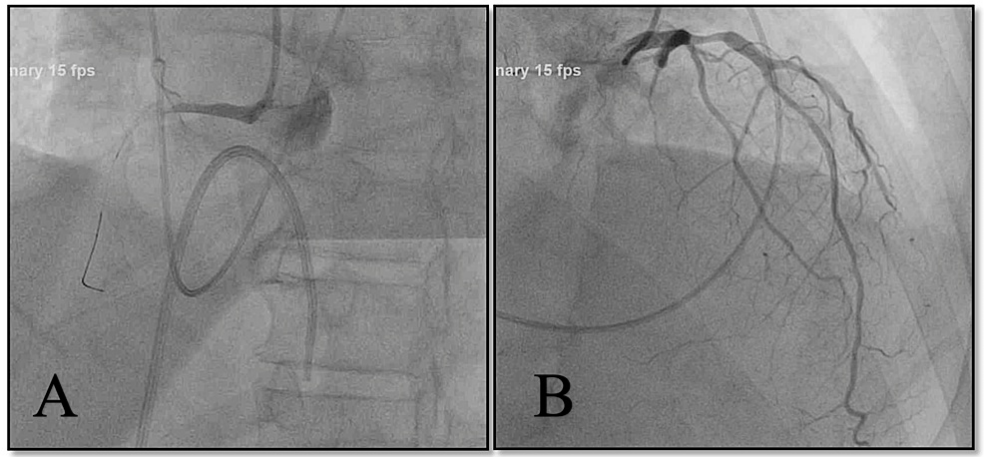
Coronary angiogram showing (A) the right coronary artery with 100% occlusion of the proximal part, and (B) stenoses of the left anterior descending artery and the left circumflex artery.

During an urgent cardiac catheterization (Figure 1), severe triple vessel disease was found: the left anterior descending artery (LAD) showed 60% proximal stenosis, 50% mid stenosis, and distal subtotal stenosis at the apex. Additionally, the D1 bifurcating vessel was completely stenotic in the proximal branch, with left-to-left collaterals. Proximally, the left circumflex (LCx) was patent, while the distal LCx showed 99% focal stenosis. There was 100% proximal occlusion of the right coronary artery (RCA), with a left-to-right collateral branch to the distal RCA. A left ventricular ejection fraction of 35% was estimated. Despite efforts to revascularize the diseased coronary vessels, it was unsuccessful; as a result, he was transvenously paced. Fluids and vasopressors (norepinephrine) were given for cardiogenic shock (mean arterial blood pressure was below 50 mmHg). An X-ray of the chest showed mild infiltration and atelectasis in the right lower base of the lung. Routine polymerase chain reaction (PCR) for SARS-CoV-2 virus was positive.

Other laboratory findings were suggestive of DIC and multiorgan failure (Table 1). DIC prevented the initiation of dual antiplatelet therapy. Due to persistent oliguria and failure to respond to furosemide, the patient required emergency continuous renal replacement therapy (CRRT). Prophylactic anticoagulation was not started as the patient was scheduled to have vascular access for the CRRT. Ten units of cryoprecipitate were administered before placement of the dialysis access. He was also administered parenteral vitamin K. On day 2 of the hospital stay, his hemoglobin concentration dropped from 11.2 g/dL to 9.0 g/dL. He was transfused with one unit of RBCs. He remained dependent on vasopressor support. The poor liver and renal function prevented the administration of antivirals for COVID-19 infection. Repeated clotting episodes at the dialysis access despite flushing with alteplase made continuation of CRRT difficult. On the third day of hospitalization, prophylactic anticoagulation with heparin was started after discussing the risks with the healthcare proxy. The patient developed cardiac arrest, cardiopulmonary resuscitation attempts were futile, and he unfortunately expired.

## Discussion

DIC results from systemic hemocoagulation in which fibrin clots and microthrombi are formed in the microcirculatory vessels, resulting in fibrinolysis and exhaustion of anticoagulant activity. Multiple organ failure is associated with DIC, caused by impaired microcirculation [20-23]. A study by Tang N et al. [24] initially reported a high prevalence of DIC in COVID-19, but subsequent studies revealed that overt DIC is uncommon [12,13,25,26]. The diagnosis of COVID-19-associated DIC requires excluding COVID-19-associated coagulopathy (CAC), which is a close differential diagnosis. Most patients with CAC have a normal/elevated fibrinogen concentration, a normal or mildly reduced platelet count and prothrombin time [24,25,27,28]. Table 2 compares diagnostic criteria for DIC and CAC.

**Table 2:** Criteria for diagnosis of DIC and CAC. DIC: Disseminated Intravascular Coagulation; ISTH: International Society of Thrombosis and Hemostasis; CAC: COVID-19-associated coagulopathy; INR: International normalised ratio; PT: Platelets.

ISTH DIC criteria [P]: ≥5 points	CAC criteria [O]: ≥2 of the following
*Platelet count, × 10^9^ /L. => * < 100 =1 < 50 = 2	Platelet count < 150 x 10^9^
D-dimer increased	D-dimer elevation > 2 X ULN
PT Prolonged ≥3 but => < 6s=1 ≥ 6 s = 2	PT prolonged >1s or INR > 1.2
Fibrinogen => ≤1.0g/L = 1	Presence of micro and/or macrovascular thrombosis

For the virus to enter endothelial cells, it must bind protein S to the angiotensin-converting enzyme type 2 [29]. As a consequence, inflammation occurs within the endothelium, damaging its cells and disrupting its anticoagulant function, thereby increasing the propensity for thrombosis [30]. Coagulopathy is associated with concurrent disorders within the vWF-ADAMTS13 axis, which are linked to platelet activation and coagulation cascades [31]. There is an interaction between the innate response mechanisms of the body, the coagulation system, and the complement system that causes thromboembolism and the formation of blood clots in large and small vessels [32]. The virus does not appear to have a procoagulant effect; instead, it is thought to trigger an inflammatory reaction in the host that leads to consumptive coagulopathy [33]. The occurrence of coagulopathy has been demonstrated by several parameters such as prothrombin time, activated partial thromboplastin time, D-dimer, fibrinogen, erythrocyte sedimentation rate (ESR), C-reactive protein (CRP), thrombocytopenia, and IL-6 levels which were abnormal, particularly in ICU patients [33-39]. It has been shown that elevated D-dimer levels are associated with an increased risk of mortality [10,34,40]. DIC generally leads to systemic thrombosis; however, there are few cases in which coronary vessels are affected [14-18]. In the literature, there are few reports of COVID-19-associated DIC presenting as STEMI [18,19]. It is unclear how DIC contributes to coronary thrombosis. Patients with DIC have been noted to have low levels of protein C and anti-thrombin-III, the body's natural anticoagulants [41-43]. Thrombosis of the coronary, jugular, and mesenteric vessels has been associated with reduced levels of these natural anticoagulants [44,45]. In DIC, thrombin production and fibrinolysis are ongoing processes, and the resolution of these abnormalities relies on removing the inciting stimuli. An individualized approach is often recommended for managing DIC based on the underlying disease and the presence of bleeding or thrombotic complications. In patients with the bleeding form of DIC, replacement therapy with antifibrinolytic agents and/or platelets will be beneficial, while patients with predominantly thrombotic DIC should undergo anticoagulation therapy [46,47]. Thromboprophylaxis with low-molecular-weight heparin is recommended for the thrombotic type of DIC, except if there is bleeding or the platelet count is less than 30x109/L [48]. Although our patient had predominantly thrombotic DIC, he was not immediately anticoagulated due to the planned invasive procedures (vascular access for CRRT) and concerns about ongoing internal bleeding evidenced by decreased hemoglobin. Irrespective of the form of DIC, the single-most critical goal in management is to treat the underlying condition contributing to continuous coagulation and thrombosis [49].

Due to thrombocytopenia and an increased risk of bleeding, DIC can complicate the management of STEMI. Antiplatelet and antithrombotic agents are contraindications in this context. Furthermore, it is uncertain whether invasive revascularization will be successful, given the high mortality rate reported in the literature [14-16,18-19]. In our case, the patient underwent an urgent cardiac catheterization that was unsuccessful. The DIC prevented him from receiving dual antiplatelet and antithrombotic treatment. The patient developed multiorgan failure, making him ineligible for COVID-19 anti-vials. Despite using alteplase to flush the lines, the vascular access for CRRT repeatedly became blocked by blood clots. According to previous studies (Table 3), the outcomes of DIC in COVID-19 are often poor as the mortality rate is high. We strongly think our patient had DIC, likely from COVID-19, and not CAC, because he had an ISTH score of 8 with reduced fibrinogen levels, significant thrombocytopenia, and prolonged coagulation times, which are usually not seen in CAC. It is important to note that the absence of overt bleeding does not rule out DIC since diffuse bleeding occurs in only 5-12% of patients, particularly those with vascular abnormalities or leukemias. The thrombotic form of DIC is more common, occurring in up to 40% of patients [46,50].

**Table 3:** Proportion and mortality of hospitalized patients with COVID-19 who developed DIC. DIC: Disseminated intravascular coagulation.

Author (year)	The proportion of hospitalized COVID-19 patients with DIC	Mortality
Tang N et al. (2020) [10]	16/183 (8.7%)	71.4%
Al-Samkari H et al. (2020) [26]	3/400 (0.75%)	Not stated
Helms J et al. (2020) [12]	0	0
Lodigiani C et al. (2020) [13]	8/375 (2.1%)	88%

## Conclusions

COVID-19-associated DIC can cause coronary vessel thrombosis, leading to MI. As the pandemic continues, a high index of suspicion for DIC, especially the thrombotic form, must be maintained in hospitalized patients with COVID-19 infection. DIC may complicate the management of STEMI, and outcomes are particularly poor in the setting of COVID-19 infection. A multi-disciplinary approach is often needed in the management of these patients.
